# Cricket antennae shorten when bending (*Acheta domesticus* L.)

**DOI:** 10.3389/fphys.2014.00242

**Published:** 2014-06-26

**Authors:** Catherine Loudon, Jorge Bustamante, Derek W. Kellogg

**Affiliations:** ^1^Department of Ecology and Evolutionary Biology, University of California-IrvineIrvine, CA, USA; ^2^Department of Ecology and Evolutionary Biology, University of KansasLawrence, KS, USA

**Keywords:** cricket, antenna, bending, cuticle, insect, *Acheta domesticus*

## Abstract

Insect antennae are important mechanosensory and chemosensory organs. Insect appendages, such as antennae, are encased in a cuticular exoskeleton and are thought to bend only between segments or subsegments where the cuticle is thinner, more flexible, or bent into a fold. There is a growing appreciation of the dominating influence of folds in the mechanical behavior of a structure, and the bending of cricket antennae was considered in this context. Antennae will bend or deflect in response to forces, and the resulting bending behavior will affect the sensory input of the antennae. In some cricket antennae, such as in those of *Acheta domesticus*, there are a large number (>100) of subsegments (flagellomeres) that vary in their length. We evaluated whether these antennae bend only at the joints between flagellomeres, which has always been assumed but not tested. In addition we questioned whether an antenna undergoes a length change as it bends, which would result from some patterns of joint deformation. Measurements using light microscopy and SEM were conducted on both male and female adult crickets (*Acheta domesticus*) with bending in four different directions: dorsal, ventral, medial, and lateral. Bending occurred only at the joints between flagellomeres, and antennae shortened a comparable amount during bending, regardless of sex or bending direction. The cuticular folds separating antennal flagellomeres are not very deep, and therefore as an antenna bends, the convex side (in tension) does not have a lot of slack cuticle to “unfold” and does not lengthen during bending. Simultaneously on the other side of the antenna, on the concave side in compression, there is an increasing overlap in the folded cuticle of the joints during bending. Antennal shortening during bending would prevent stretching of antennal nerves and may promote hemolymph exchange between the antenna and head.

## Introduction

**Figure 1:**
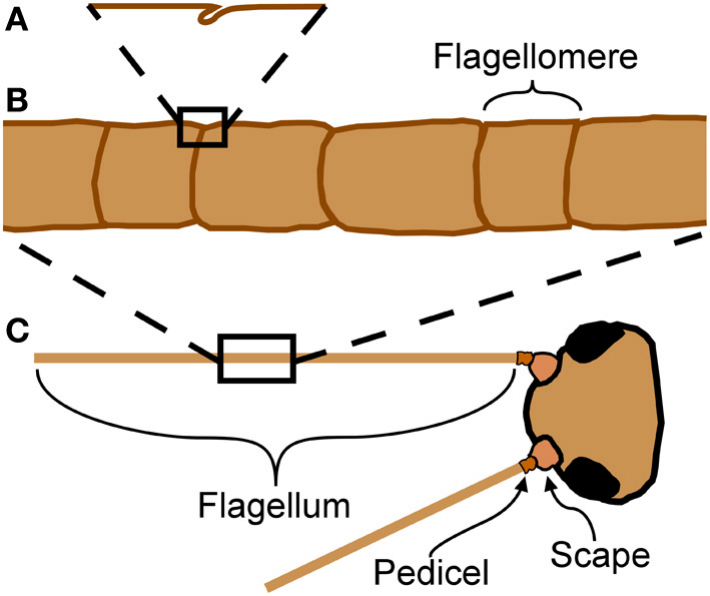
**Anatomy of cricket antennae. (A)** Flagellomeres are separated by joints of folded cuticle. **(B)** Flagellum of cricket antenna with large number of flagellomeres. **(C)** An insect antenna has three true segments: the scape, the pedicel, and the flagellum.

Arthopods are encased in an exoskeleton made of cuticle. Insect cuticle can be quite stiff but also varies between insects and locations on the body; published measurements of stiffness (Young's modulus) vary over about seven orders of magnitude from 1 kPa to 20 GPa (Vincent and Wegst, 2004). Flexible joints are necessary to allow relative movement between stiff parts of an exoskeleton. In insects (and other arthropods), a joint may be formed from thinner or more flexible cuticle (Hepburn and Chandler, 1976), usually associated with a fold (invagination) in the cuticle (Snodgrass, 1935). A planar fold (Figure 1A) will tend to act like a simple hinge joint, allowing rotation at this localized area of lower stiffness (Winder et al., 2009). A planar fold may wrap around a cylindrical appendage, allowing bending in any direction at the joint formed by this cylindrical fold (Figure 1B).

Insect antennae are sensory appendages that bend in response to external forces, affecting sensory input from the mechanosensors. This mechanosensory information is used for diverse functions such as flight control (Gewecke and Heinzel, 1980; Heinzel and Gewecke, 1987), wall-following (Camhi and Johnson, 1999), and hearing in mosquitoes (Göpfert et al., 1999). Antennae of some insects, such as the domestic cricket (*Acheta domesticus* L.), are long and filiform with a very large number of subsegments (called flagellomeres) separated by joints (Figure 1C). These joints appear to be simple folds, and do not have muscular attachments. Muscles do not extend into the flagellum (the longest and terminal segment, Figure 1C) in insects, although they do for two groups of entognathous hexapods, the Collembola and Diplura (Chapman, 1998).

**Figure 2:**
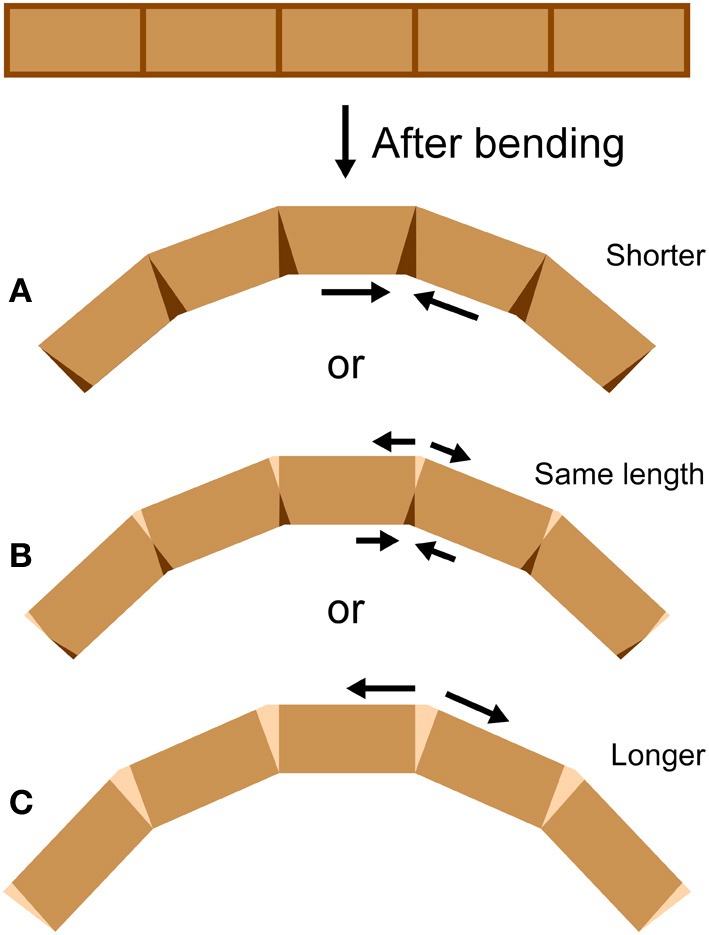
**Bending at joints.** If a cylindrical structure only bends at joints, it can **(A)** shorten, **(B)** stay the same length, or **(C)** lengthen during bending, depending on the mechanical behavior of the joints (length measured along the central midline). At the joints, darkened areas indicate shortening (such as by cuticle folding or compressing) and lighter areas indicate lengthening (such as by cuticle unfolding or stretching).

**Figure 3:**
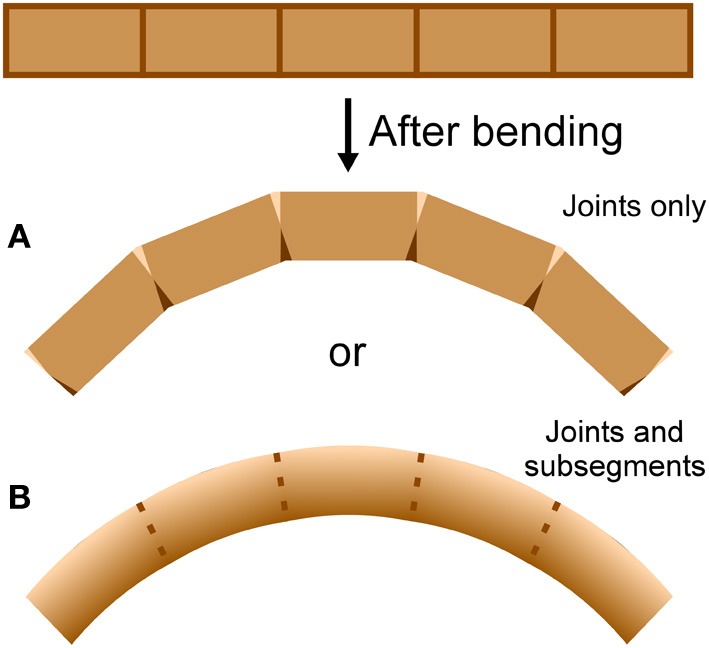
**Bending location.** A cylindrical structure with apparent joints could actually bend at **(A)** the joints only or **(B)** the joints and the flagellomeres, depending on the mechanical behavior of the joints and the flagellomeres. Darker areas indicate shortening (such as by cuticle folding or compressing) and lighter areas indicate lengthening (such as by cuticle unfolding or stretching).

An antenna may be considered as a cantilever beam because it is attached at one end and extends into space (Loudon, 2005). As a beam (or antenna) bends, its convex side will be in tension and its concave side in compression (Vogel, 2013). Because an antenna is a thin-walled structure, the cross-sectional shear that is generated during bending and is of lowest magnitude at the upper and lower surfaces (Denny, 1988, p. 199) can be ignored. If a bending structure has comparable properties in tension and compression, the structure will tend to lengthen on the convex side and shorten on the concave side, with a centrally-located neutral axis of unchanging length down the middle (Wainwright et al., 1976; Vogel, 2013). It is unknown whether bending antennae follow such a simple pattern; a bending antenna could hypothetically lengthen or shorten overall during bending if it is stiffer in either tension or compression respectively, or if the material properties of the cuticle vary circumferentially (Figure 2). In addition, it is unknown whether the bending only takes place at the joints, or if the interposing flagellomeres also bend (Figure 3). In order to examine the mechanical behavior of the flagellum during bending, and therefore more insight into the inputs available to antennal mechanosensors, live crickets were constrained, their antennae were bent in a number of different directions, and micrographs were taken and analyzed.

## Materials and methods

### Experimental animals

Crickets (*Acheta domesticus* L.) were purchased in a pet store (PetCo) and kept in an insect rearing cage in an insect rearing room on a 16:8 L:D light cycle, with temperatures typically varying between 20 and 25°C. Crickets were given *ad libitum* access to water (Fluker's Cricket Quencher) and saltine crackers. Morphological measurements on detached whole antennae permanently mounted on slides were made for 30 adult crickets (15 females and 15 males). Bending measurements were made on attached antennae for an additional 15 adult crickets (eight females and seven males).

### Preparation of images of antennae

#### Whole antennae mounted on glass slides

Antennae were removed in their entirety still attached to small pieces of the head capsule (for orientation). Both antennae were removed from 15 adult male and 15 adult female crickets. The antennae were then placed in 50 ul microcapillary pipets to protect them and keep them straight during dehydration (through an ethanol sequence to 100% ethanol) and clearing (Hemo-De for 24 h). Antennae were then removed from the pipets and mounted onto glass slides using mounting medium (Permount). Pictures of the antennae were recorded using a Panasonic WV-CL700 video camera mounted on a dissecting microscope (Zeiss Stemi SV-6). A composite of 11 adjacent (partly overlapping) still images was necessary to encompass the entire length for each individual antenna, allowing a resolution of 9 microns per pixel. Composites were generated by translating and rotating the individual images manually; partly overlapping images were made slightly transparent temporarily to guide the overlay process within Canvas (ACDSee Systems).

#### Comparison of straight and bent antennae attached to live crickets

Each antenna, still attached to a live cricket, was positioned in a horizontal plane and photographed from above using a camera (Canon EOS Rebel XT) mounted on a dissecting microscope (Zeiss Discovery V20). A short part of each antennal flagellum was viewed under high magnification (50–225×) for measurement of flagellomere size and joint position. Images were saved as JPG files, 3456 × 2304 pixels, with resolution of 0.8 microns at 50× and 0.18 microns at 225× (size of one pixel). The average length of these analyzed sets of flagellomeres was 1.3 mm (range 0.5–2.1 mm) and had an average of 9 adjacent flagellomeres (range 4–16) (*n* = 29 sets of flagellomeres from 15 antennae; each antenna was used for two bending directions). The flagellomeres were photographed three times: first with the flagellum straight, then bent, and then re-straightened. The re-straightened flagellum served as a control, allowing evaluation of any changes in morphology of the antenna caused by bending. Flagellomere length is highly variable along a flagellum, and therefore patterns in flagellomere length were used to match individual flagellomeres when comparing bent with straight antennae.

#### Scanning electron microscopy of flagella

Low vacuum SEM imaging was performed on a FEI Quanta 3D FEG Dual Beam SEM (FEI, Hillsboro, Oregon). Antennae were removed from adult male and female crickets and mounted either curved or straight on stubs using carbon tape or copper tape without further processing. Images were attained at a pressure of 0.6 mbar and 5 kV with water as the ionizing gas.

### Bending of antennae

#### Restraining crickets

**Figure 4:**
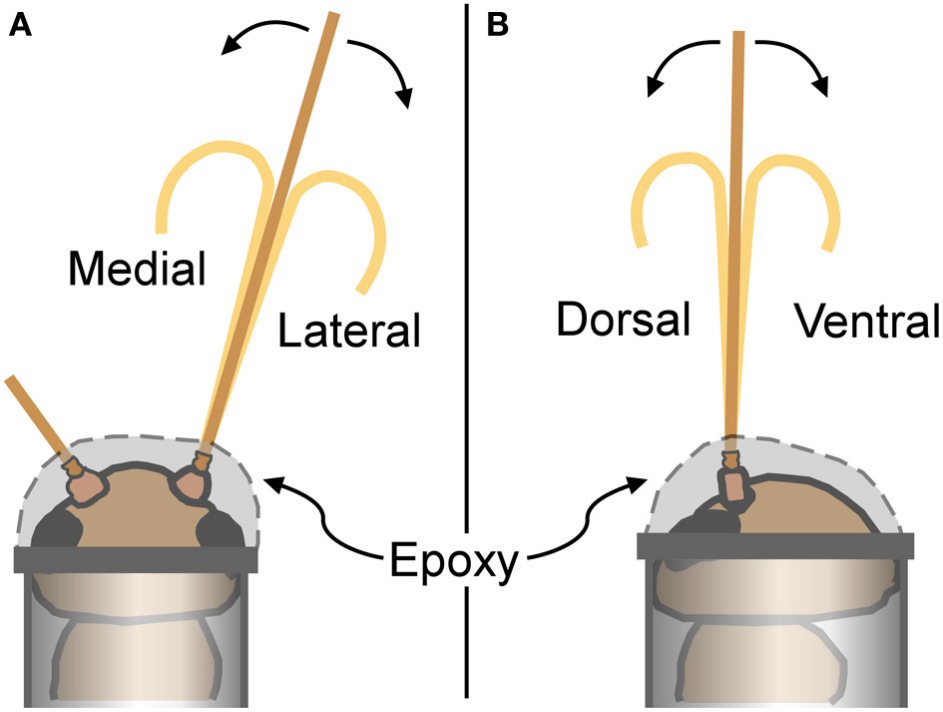
**Cricket restraints and bending directions.** For antennal bending measurements, crickets were placed into plastic tubes, and attached with epoxy that secured the base of the antenna (epoxy covered the scape, pedicel, and the first few flagellomeres). **(A)** Antennae bent in a medial or lateral direction were viewed from a dorsal perspective as illustrated. **(B)** Antennae bent in a dorsal or ventral direction were viewed from a lateral perspective.

Individual crickets were restrained by placing them into microcentrifuge tubes (0.6 ml size for the males, and 1.5 ml for the females; Fisherbrand polypropylene) after exposure to carbon dioxide for about a minute to inhibit movements during handling. After insertion into the tube and before the cricket started moving again, the head of the cricket was firmly attached to the opening of the centrifuge tube with 5 min epoxy (Ace Quick Set Epoxy). One antenna was selected for bending measurements, and its base (scape and pedicel) was encased in epoxy including the pedicel-flagellum joint, leaving the flagellum available for bending (Figure 4). The other antenna was secured out of the way by epoxy. The closed tip of the centrifuge tube was cut off in order to expose the abdomen and thorax of the restrained cricket to room air. After securing the cricket, leg movements were visible through the centrifuge tube wall indicating cricket viability.

#### Mechanical manipulation of antennae

Antennae were bent in a total of four different directions: dorsal, ventral, medial, or lateral (Figure 4). Only one antenna was used for each cricket (alternating left and right antenna between individuals). Any antenna was bent in two different directions (such as dorsal and medial), with the order randomized. All four bending directions were not used for each individual antenna to avoid breakage caused by the repeated handling. There was a sample size of 3–5 antennae for each gender-direction combination.

An antennal flagellum was gently bent into a “u-shape” by bringing the ends of two rods (glass capillary tubes) toward each other until a small antennal loop was formed between the ends that included the selected set of flagellomeres. Antennae were not bent to the point of mechanical damage, plastic deformation, or kinking. Bending directions were alternatively assigned to cricket antennae, and females and males were alternated. Bending took place in a horizontal plane at a right angle relative to the microscope view; the cricket was rotated to have the appropriate side up (dorsal, ventral, left, or right) for the desired bending direction (Figure 4). Images were taken as described above.

#### Experimental uncertainty in bending direction

In order to estimate the experimental uncertainty in setting the orientation of the head, and therefore the bending direction, we used the position of the cricket's eyes to identify the true dorsal. Restrained crickets were positioned in the holder with dorsal, ventral, left, or right sides oriented upward. We took “heads-on” pictures of each cricket head (Sony Handycam HDR-CX100) from a horizontal direction, with a plumb line in view to establish a true vertical, and compared the angle of a line segment connecting the two eyes with the true vertical. The actual angle was measured from the images to the nearest degree for 3 crickets each at four orientations. The average deviation from the intended angle was 0.8° (*n* = 12); the average of the absolute values of the deviations from the intended angle was 7.2° (*n* = 12).

### Image analysis

#### Flagellomere morphology

**Figure 5:**
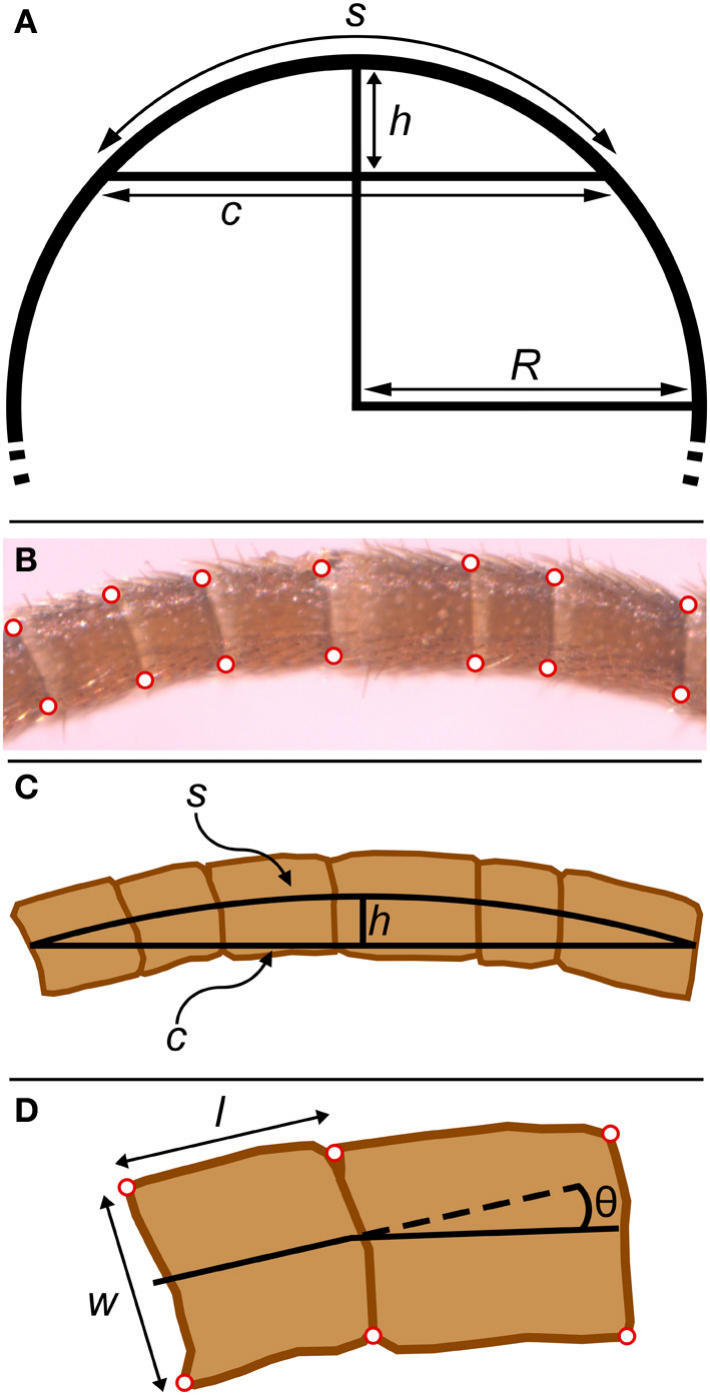
**Geometrical parameters in bent antennae. (A)** The relationship between chord (*c*), arc length (*s*), sagitta (*h*), and radius (*R*) in a circle. The sagitta is the line segment that starts at the center of the chord, is orthogonal to the chord, and terminates where it intersects with the circle. **(B)** Micrograph of bent antenna with corners digitized. **(C)** Equivalent geometrical features for the experimental part of a bent flagellum. The chord (*c*) was approximated as the line segment joining the two terminal midpoints of the experimental section of the flagellum. The radius of curvature (*R*) was calculated from the chord (*c*) and sagitta (*h*) as explained in the text. **(D)** The four corners of each flagellomere (red) were digitized. Lengths (*l*) and widths (*w*) for all flagellomeres were calculated from these points. The angle between adjacent flagellomeres was calculated from the angle between line segments connecting the midpoints of the digitized points.

The four corners of each flagellomere on the micrographs were digitized manually using Didger4 (Golden Software, Golden CO), thereby approximating each flagellomere as a trapezoid (Figure 5). The digitized coordinates were used to calculate the lengths of the sides of the trapezoid using the distance formula (Pythagorean theorem). The lengths (*l*) and widths (*w*) of flagellomeres were calculated from the average of the two relevant sides (parallel or perpendicular to the longitudinal axis of the flagellum, respectively) (Figure 5D). The radius of the flagellum (*r*) at a specific flagellomere will be half of the width (*w*/2).

For the 60 whole antennae permanently mounted on glass slides (both flagella from 15 adult males and 15 adult females), the corners of all flagellomeres on the composite images were digitized for both flagella. This allowed us to quantify the total number of flagellomeres on each antenna and also how flagellomere length and width varied along the length of flagella. Using a composite image had negligible effect on measurements of individual flagellomere length and width, because each individual component image contained several flagellomeres, and any flagellomere could be identified in intact form in some image. Left and right antennae from an individual cricket were not always the same length; the tips of cricket antennae are often broken off. For calculating the average individual flagellomere widths and lengths (i.e., average for flagellomere 1, average for flagellomere 2, etc.), data from only one antenna (the longer one) from each cricket was used. For calculating the average length of flagellomeres for each cricket, the first 50 flagellomeres from both antennae for each cricket were used and averaged together for each cricket.

For the 15 antennae involved in bending measurements, only a set of flagellomeres in the bent portion of the antennae were digitized. In addition to measurement of the lengths and widths of the flagellomeres, the angles between flagellomeres were calculated from line segments connecting the centers of adjacent flagellomeres (Figure 5D). These angles will be influenced by flagellomere shape irregularities. Therefore, the change (difference) in these angles between bent and straight antennae was used to estimate bending angles.

The experimental error of the digitized lengths and widths has a number of contributing sources; the overall error or uncertainty can be estimated by comparing repeated measurements. Repeated measurements exist for flagellomeres in antennae used in the bending measurements. These antennae were photographed 3 times: straight, bent, and then re-straightened. The average difference in length measured for the same flagellomeres in the straight and re-straightened configuration was 0.2 microns, with a standard deviation of 4.96 microns (*n* = 269 flagellomeres).

#### Estimate of radius of curvature for antennae after bending

The radius of curvature (*R*) for the photographed sections of curved (bent) antennae were calculated by approximating each curved set of flagellomeres as the arc of a circle (s), using the relationship between a chord (*c*), a sagitta (*h*), and the radius (*R*) of a circle (Beyer, 1981, p. 125) (Figure 5). The chord was approximated as the line segment joining the two terminal midpoints of the experimental section of the flagellum. The sagitta was slightly more complicated to calculate from the digitized points of the flagellomeres: the sagitta will be part of a line that bisects the chord (Figure 5C), and therefore can be identified from (1) the equation of the line that bisects the chord (calculated from the chord endpoints) and (2) the line segment that connects the midpoints of the edges of the central flagellomere. From the lengths of the chord and the sagitta, the radius of curvature of that experimental section of the flagellum can be estimated from:

$$R=\frac{c^{2}+4h^{2}}{8h}$$

Note that *R* is measured to the midline of the experimental section. The two edges (convex and concave) of the curved experimental section may be visualized as parts of two concentric circles that are 2*r* apart, which means that the ratio of the circumferences of the outer to inner circles is (*R* + *r*)/(*R* − *r*), which will also be the ratio of the lengths of the convex to concave edges.

#### Comparison of flagellomere shapes in straight and bent antennae

In order to determine whether bending occurs only at the joints between flagellomeres, or also in the flagellomeres themselves, the shapes of the individual flagellomeres were examined in micrographs of the experimental section of the flagellum. The irregular perimeters of the flagellomeres were traced using a polyline tool (Canvas 12, ACDSee Systems) for both the straight and bent sections. The tracings were digitally overlaid, translated, and rotated manually until they were aligned.

#### Estimate of volume

The volume of the experimental section of a flagellum when straight or bent was estimated using a formula for the volume of a right circular cylinder or partial torus, respectively. The volume of a right circular cylinder (*V*_cylinder_) of length (*L*) and radius (*r*) is *V*_cylinder_ = π*r*^2^*L*. Therefore, the volume of the straight experimental sections were calculated from this equation using half of the average width of the flagellomeres for *r*, and the summed length of the straight flagellomeres for *L*. The volume of a torus (*V*_torus_) of radius of curvature *R* and “revolving” radius *r* is *V*_torus_ = 2π^2^*Rr*^2^, and therefore the volume of a partial torus can be estimated by multiplying *V*_torus_ by the ratio of the arc length (*s*) to the complete circumference (2π*R*) (both measured down the midline),

$$V_{partial\;torus}=2\pi^{2}Rr^{2}\frac{s}{2\pi R}=\pi r^{2}s$$

(note that this result is identical to *V*_cylinder_ when *s* = *L*). Alternatively, if the length of the convex side is known, the volume of a partial torus can be estimated by multiplying *V*_torus_ by the ratio of the convex length to the complete circumference (2π(*R* + *r*)) (both measured along the convex perimeter):

$$\begin{array}{l} V_{partial\;torus}=2\pi^{2}Rr^{2}\frac{\left(\text{summed length on convex side}\right)}{2\pi \left(R+r\right)}\\ \;=\frac{\pi Rr^{2}\left(\text{summed length on convex side}\right)}{R+r} \end{array}$$

The volume of the curved (bent) experimental sections of the flagella were calculated from the formula above.

### Statistics

All statistical tests were performed using SAS Version 9.3 (Cary, NC).

## Results

### Morphology of flagella

**Figure 6:**
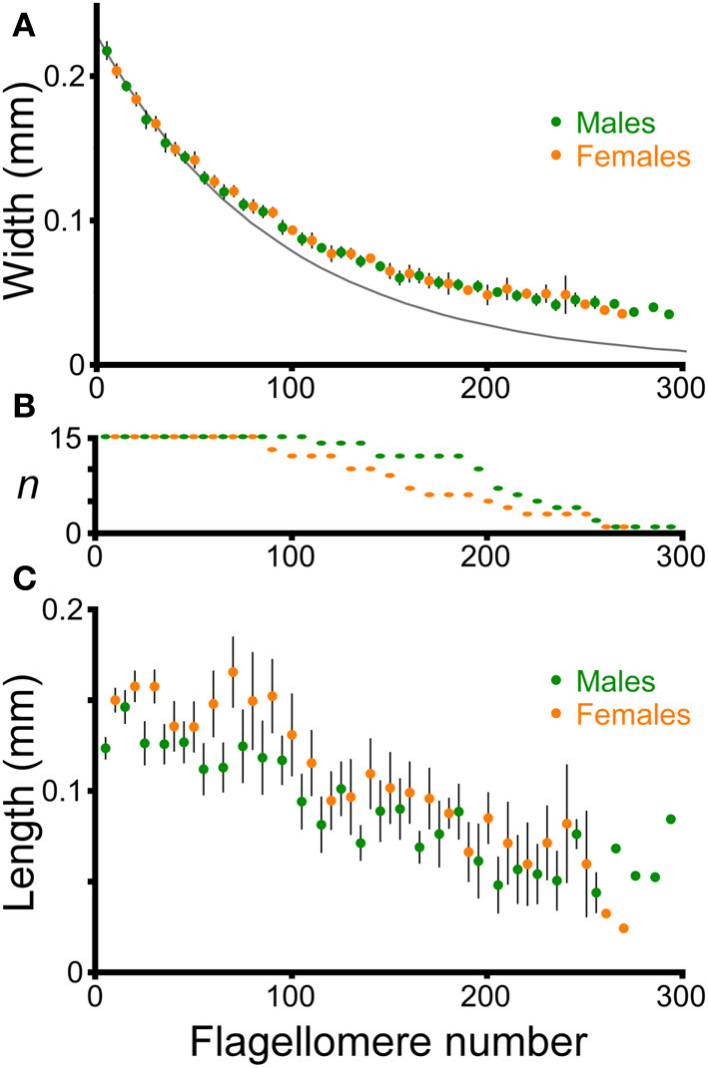
**Morphology of flagellomeres.** Flagellomeres are numbered from proximal to distal along the flagellum. One antenna was used from each of 15 adult males and 15 adult females. **(A)** Width of individual flagellomeres (mean ± 2 s.e.m.). Only the data from every tenth flagellomere are graphed for clarity, and are staggered to facilitate comparison (males: 5th, 15th, 25th, etc. flagellomeres; females: 10th, 20th, 30th, etc.). The dark gray line is the simple exponential fit for the averages of the first 50 flagellomeres for both males and females: *y* = e^∧^( − 0.010744*x* − 1.46984), where *y* = width in mm, and *x* is flagellomere number (*r*^2^ = 0.97). **(B)** Sample size of flagellomeres used for each point (shorter antennae have fewer flagellomeres). **(C)** Length of individual flagellomeres (mean ± 2 s.e.m.). Only the data from every tenth flagellomere are graphed for clarity, and are staggered, as in **(A)**.

The flagella decreased in width (diameter) smoothly from the proximal to the distal end in both males and females (Figure 6A). The relationship between width and flagellomere number was approximately exponential (log(width) varied linearly with flagellomere number), but did show small changes along the length of a flagellum. For example, the exponential equation fit to the first 50 flagellomeres provides an increasingly poor fit for more distal flagellomeres (Figure 6A). Therefore to make comparisons between antennae, the intercepts and slopes of log-linear regressions using only the most proximal 50 flagellomeres were used to quantify width and taper. All 30 antennae had at least 50 flagellomeres (Figure 6B). The intercepts and slopes from the left and right antennae were averaged for each cricket. There was no significant difference between males and females in flagellum width (intercepts of log-linear regressions were not significantly different, ANOVA, *P* = 0.19, *n* = 30) or in flagellum taper (slopes of log-linear regressions were not significantly different, ANOVA, *P* = 0.39, *n* = 30). Flagella are approximately circular in cross-section; pairwise comparisions of diameter measured for the same flagella from both a dorsal and a lateral view differed by an average of only 3% (*n* = 9, average diameter at measured flagellomere was 0.09 mm). Therefore no adjustment for orientation was necessary in calculating diameter.

The length of adjacent flagellomeres was highly variable along the flagellum in both males and females (Figure 6C), although there was a trend to shorter flagellomeres in the distal direction. To simplify comparisons between males and females, the average length of the first 50 flagellomeres was used as a representative morphological parameter for each antenna (for each individual, the left and right numbers were averaged). Although there was a slight trend toward longer flagellomeres on average in females (144 microns for females, 135 microns for males; averages of first 50 flagellomeres, *n* = 15 for both males and females), this was not significantly different (ANOVA on the average length of the first 50 flagellomeres, *P* = 0.07, *n* = 30). The striking variability in length of adjacent flagellomeres is similar to what has been reported for female stick insect antennae (Dirks and Dürr, 2011).

### Antennae shorten when they bend

**Figure 7:**
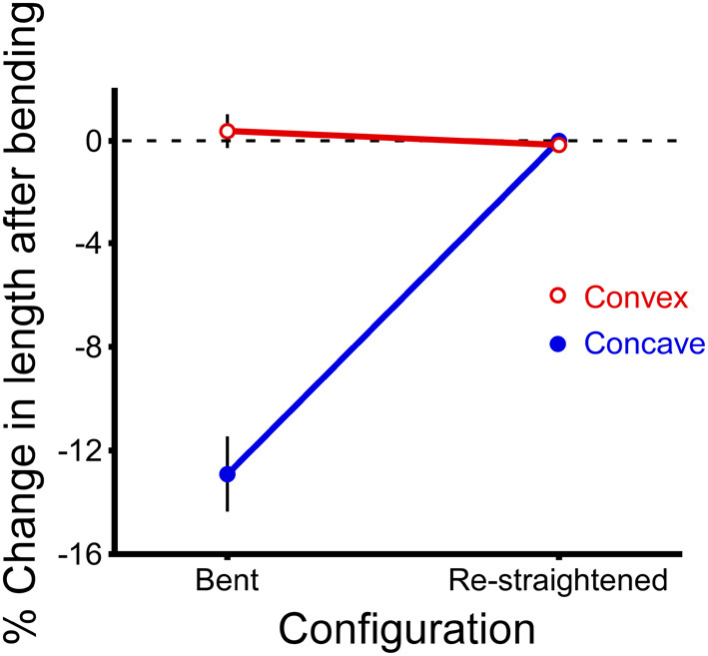
**Change in length after bending.** Lengths of the experimental sections of antennae were calculated for both the convex and concave sides of each antenna by summing the digitized lengths of the adjacent flagellomeres before bending, after bending (“bent”), and after re-straightening (“re-straightened”). The percent change in length is calculated relative to the length of the initial (straight) section before bending. Data have been combined for both sexes and all four directions of bending because they are not significantly different from each other (*n* = 29 bending measurements, *n* = 15 antennae, each antenna bent in two directions, mean ± 2 s.e.m.).

**Figure 8:**
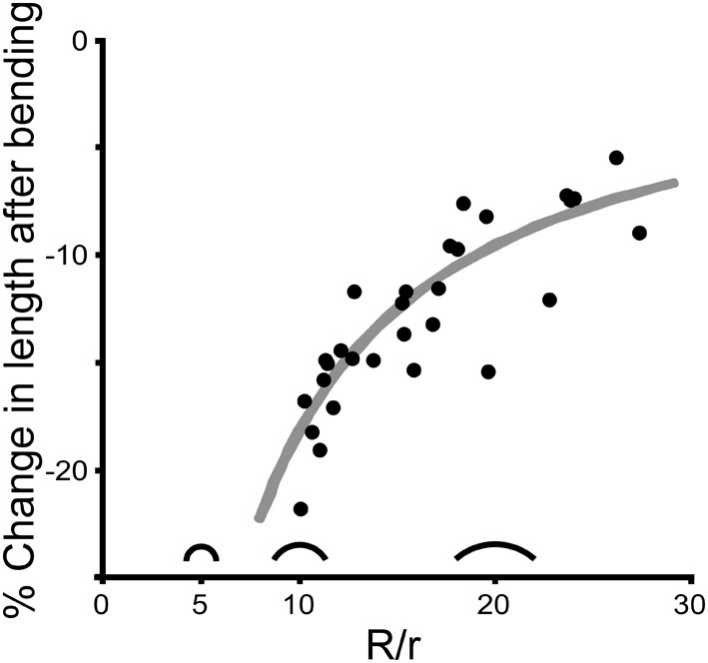
**Change in length is affected by curvature.** Each point shows the percent decrease in length on the concave side for one experimental section of an antenna caused by bending one antenna in one direction (*n* = 29). The amount of bending is reported as dimensionless curvature (*R*/*r* where *R* is the radius of curvature and *r* is the average radius of the bent section of the antenna), with three silhouettes illustrated as examples (*R*/*r* = 5, 10, and 20). The gray line represents the theoretical change in length on the concave side of a cylinder that is bent assuming that both the length of the convex side and the diameter remain unchanged during bending.

When a cricket antenna was bent into a tight loop, the bent portion of the flagellum was measurably shorter than when it was straight. Specifically, the summed lengths of the flagellomeres on the concave side of the antenna (the side in compression) were significantly shorter than when straight (paired *t*-test, *P* < 0.0001, *n* = 29) (Figure 7). In contrast, the summed lengths of the flagellomeres on the convex side of the antenna (the side in tension) were not significantly different than when straight (paired *t*-test, *P* = 0.39, *n* = 29) (Figure 7). The lengths of re-straightened antennae were not significantly different from the original straight lengths on either the convex or concave side (paired *t*-test, *P* = 0.34 for convex side, *P* = 0.93 for concave side, *n* = 29 for each) (Figure 7). This pattern, that only the concave side of the antennae exhibited a reversible change in length during bending, was the same for both males and females, and was the same for all four directions of bending; a two-way ANOVA did not show a significant effect of either sex (*P* = 0.189) or bending direction (*P* = 0.165) on the percentage change in length. The experimental sections decreased in length on the concave side by an average of 13% (range 5–22%, *n* = 29), which corresponds to a decrease of 0.17 mm. The decrease in length was greater when the bend was a sharper curve (using a dimensionless radius of curvature, *R*/*r*) (Figure 8). The average radius of curvature (*R*) was 0.72 mm (range 0.43–0.95 mm, *n* = 29), the average radius of the experimental section (*r*) was 46 microns (range 34–63 microns, *n* = 29), and the average dimensionless radius of curvature (*R*/*r*) was 16 (range 10–27, *n* = 29).

### Bending occurs at joints

**Figure 9:**
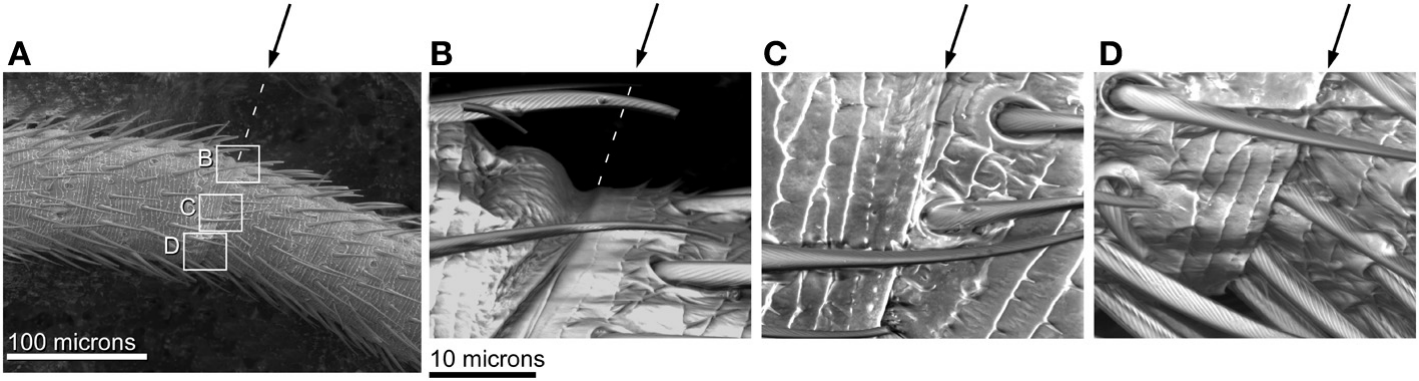
**SEM images of joints in bending antenna.** Arrows indicate joint. **(A)** A bent flagellum from an adult male. Rectangles denote parts of joint shown in higher magnification in **(B–D)**. **(B)** Closer view of joint on convex side of bent flagellum (in tension) showing slight unfolding at joint. **(C)** Closer view of middle of joint in bent flagellum; angle between adjacent flagellomeres shown by surface sculpture. **(D)** Closer view of joint on concave side of bent flagellum (in compression) showing fold touching base of hairs.

Superimposing digital tracings of the same flagellomeres in straight and bent antennae revealed that bending occurred only at the joints, and that the shortening on the concave side was caused by the increasing overlap of the cuticle at the joint. That is, some of the cuticle that had been externally visible in the straight antenna folded into the joint and was no longer visible after bending (Figure 9). It was also clear that the diameters (widths) of the flagellomeres did not change during bending. For the applied degree of bending (average radius of curvature of 0.72 mm), and the average length of a single flagellomere (0.14 mm), if the flagellomeres themselves were bending in addition to the joints, a deflection of 3 microns would be expected for the center of the flagellomere. Such a deflection, which was greater than our resolution of measurement (0.8 microns), was not observed.

A flagellum bends in a continuous smooth curve despite being composed of flagellomeres of varying length, which means that the joints are irregularly spaced. The angle between flagellomeres after bending was 9° on average (210 joints on the 29 experimental sections). Occasionally adjacent flagellomeres would appear to be “stuck” together and not show bending. This occured in 11% of the joints observed (24 out of 210 joints on the 29 experimental sections). These non-bending joints tended to be between shorter flagellomeres.

### Volume is estimated to decrease during bending

If the length of a bending antenna decreases, and the diameter stays the same (see above), the overall volume will decrease. A decrease in antennal volume could force the fluid (hemolymph) inside the antenna to leave the antenna and enter the head. A bending cylinder that forms a partial torus in which the outer perimeter (on the convex side) remains constant during bending, will experience a decrease in volume that is a function of the radius of curvature (*R*) and the radius of the cylinder (*r*) (see Methods for equations). The percent change in volume will simply be (see righthand side for equation in terms of dimensionless radius of curvature)

$$\frac{r}{R+r}=\frac{1}{\frac{R}{r}+1}$$

This is exactly the same formula for the percent change in length measured down the midline of the same bending cylinder. The average percent decrease in volume (and midline length) for the bending antennal sections was 6% (range 1–11%, *n* = 29). The range in percent decrease in volume was driven primarily by the experimental variation in dimensionless radius of curvature (Figure 8).

## Discussion

The mechanical properties of insect antennae are determined in part by the pattern of folds in the cuticle. The recent mechanical literature on folded structures, including “origami engineering” and “pop-up” mechanisms, illustrate the growing appreciation of the utility and even dominating influence of folds in the overall mechanical behavior of a structure (Winder et al., 2009; Schenk and Guest, 2013; Wei et al., 2013). Applications include design of deployable structures such as airbags, solar sails, and foldable maps, as well as development of lightweight but strong materials such as foams (Heimbs et al., 2009). Some of these applications have been inspired by the study of biological folding structures such as insect wings (Haas and Wootton, 1996; Haas et al., 2000) and tree leaves (Kobayashi et al., 1998, 1999). Folds can generate both heterogeneous and anisotropic stiffness in a structure, meaning that a fold can generate a localized area of less stiffness, and that the stiffness will depend on loading direction. Therefore the orientation of a fold relative to stresses will affect its mechanical behavior; for example, a planar fold will stiffen a structure when bent at a right angle to the fold, but will act as a joint (allowing rotation, a localized area of less stiffness) when bent around an axis parallel to the fold (Figure 1). Folds in a structure allow bending of the structure as a whole without the material being stretched (Cai et al., 2013).

Joints may be characterized by their degrees of freedom of movement and also by the excursion possible before hitting a mechanical stop (Wainwright et al., 1976; Alexander, 1983). In arthropod exoskeletons, some joints are formed by folds in the cuticle, which may be thinner and less sclerotized (Snodgrass, 1935, p. 54–55; Chapman, 1998, p. 425). We found in the flagellum of cricket antennae that bending did occur only at the joints or folds between flagellomeres. The folds on the cricket antennae separating flagellomeres are not very deep, and therefore as the antenna bends, the convex side (in tension) does not have a lot of slack cuticle to “unfold” (only on the order of a few microns). Therefore the convex side does not lengthen during bending. Simultaneously on the other side of the antenna, on the concave side in compression, the flagellomeres move with respect to each other in such a way that there is an increasing overlap in the folded cuticle of the joint during bending, and the mechanical stop during compression appears to be related to the edge of the fold hitting the hairs (Figure 9). This same pattern of shortening on the concave side of a bending antenna without lengthening on the convex side was seen in both males and females, and for all four orthogonal directions of bending (to the dorsal, lateral, medial, and ventral sides). Thus, the folding or unfolding behavior of the joints was qualitatively the same around the circumference of the flagellum. Therefore, this shortening behavior is not likely to be driven by circumferential variation in material properties of the cuticle. For example, if only one side of an antenna is particularly stiff in both tension and compression, the antenna would be expected to lengthen when that stiffer side was on the concave (compression) side, but shorten when that stiffer side was on the convex (tension) side. An increasing overlap of folded cuticle between flagellomeres on the concave side of a bending flagellum has been noted for crayfish antennae (Sandeman, 1989), although a crayfish flagellum bends by different amounts in different directions.

This ability to bend in any direction by a comparable amount in a cricket flagellum is in contrast to the head-scape and the scape-pedicel joint behavior (the first and second segments of the antenna, Figure 1), both of which act more like hinge joints, and have muscles controlling their movement. The head-scape joint allows movement in a vertical plane, and the scape-pedicel joint allows movement in a horizontal plane, so the combination of the two joints allows movement of the antennal tip in three dimensions (Honegger, 1981). Note that comparable bending of the flagellum in all directions does not mean that the stiffness is necessarily the same in all bending directions; forces were not measured as part of this study. Antennae were bent in a plane without twisting because this is commonly observed during normal behavior in insects with filiform antennae. The sharpest bends in cricket antennae without mechanical damage had a dimensionless curvature of about 10 (the ratio of the radius of curvature to the radius of the bending antenna). Perhaps not coincidentally, within the wire and cable industry, manufacturers guildelines sometimes suggest a minimum bend radius that corresponds to a dimensionless curvature of about 10 (depending on cable type and loading regime, no folding). Re-straightened antennae after bending were morphologically indistinguishable from the original straight antennae, indicating that they were not damaged or plastically deformed.

Although cricket antennae were observed to shorten when bending, the diameter of the flagellum was not observed to change. If the diameter is staying the same, and length decreases during bending, then the overall volume of an antenna will decrease. The inside of an antenna contains hemolymph, which flows to and from the rest of the open circulatory system at the base of the antenna. Therefore, if the internal volume of an antenna decreases during bending, hemolymph may be forced out of the antenna; bending and straightening could be associated with driving fluid out of and into the antenna respectively. While no measurements of flow in or out of antennae have been taken during bending, excessive grooming of antennae in cockroaches (*Blattella germanica*), which requires antennal bending, has been reported to lead to temporary loss of antennal turgor and “antennal collapse” (Robinson, 1996). This suggests that bending can drive hemolymph from antennae. Crickets, like many other insects, have “accessory hearts” located at the base of the antennae that are associated with the pumping of hemolymph into the antennae from the head (Pass, 2000); these “hearts” are larger in orthopterans that have longer antennae (Pass, 1991). It is unknown how the amount of flow into an antenna generated by the action of an accessory heart would compare to that generated by a straightening antenna. It is possible that the bending and straightening of antennae would be beneficial in promoting hemolymph circulation between the antennae and the rest of the body. However, the volume changes would be expected to be small with localized bending: a maximum decrease of 10% would be expected for any part of an antenna which was bending, but the non-bending part would not experience any volume change, and therefore the overall decrease in volume in the antenna as a whole would be less than 10%. Note that if bending generates fluid movement from the antennae, it follows that net fluid movement into the antennae could cause straightening, and thus provides the basis for a possible hydraulic mechanism. There is very little known about hydraulic mechanisms of morphological reconfiguration in insect antennae; however there is evidence that increasing hemolymph pressure causes the lamellae to spread in the antennae of cockchafer beetles (Pass, 1980). Because most insects do not have intrinisic musculature within the flagella of the antennae, hydraulic mechanisms are usually assumed to be responsible for any shape changes (Zacharuk, 1985; Loudon, 2009), although a different mechanism was identified for the erection of hairs on mosquito antennae (pH-mediated swelling of annuli at the bases of the hairs; Nijhout and Sheffield, 1979).

The pattern of bending will influence the forces detected by the mechanosensors on the insect antenna. There are a large number of mechanosensors in an insect antennae: many hairs and campaniform sensilla are located on the flagellum of crickets and respond to local forces or deflections (Staudacher et al., 2005). Thus, the information is presumably available to the cricket about the extent and location of any bending of the flagellum.

A structure such as a cricket antenna that bends at folded joints is a design that allows bending without stretching of the material or twisting. This bending behavior contrasts with bending in muscular hydrostats or fiber-wound cylinders which have been analyzed in other taxa (such as elephant trunks or mammalian tongues); (Kier, 2012). We found that the limited unfolding of cuticle at the joints restricts lengthening on the convex side of a bending cricket antenna, while the ability to fold inward limits motion on the concave side, with an overall shortening occurring during bending. Shortening while bending can generate hemolymph movement, and protects internal structures such as antennal nerves from being stretched during bending of an antenna.

### Conflict of interest statement

The authors declare that the research was conducted in the absence of any commercial or financial relationships that could be construed as a potential conflict of interest.

## References

[B1] Alexander R. M. (1983). Animal Mechanics. Oxford: Blackwell Scientific Publications

[B2] Beyer W. H. (1981). CRC Standard Mathematical Tables. Boca Raton, FL: CRC Press, Inc.

[B3] Cai J. Xu Y. Feng J. (2013). Geometric analysis of a foldable barrel vault with origami. J. Mech. Des. 135, 1–6 10.1115/1.4025369

[B4] Camhi J. M. Johnson E. N. (1999). High-frequency steering maneuvers mediated by tactile cues: antennal wall-following in the cockroach. J. Exp. Biol. 202, 631–643 9929464 10.1242/jeb.202.5.631

[B5] Chapman R. F. (1998). The Insects: Structure and Function. Cambridge: Cambridge University Press 10.1017/CBO9780511818202

[B6] Denny M. W. (1988). Biology and the Mechanics of the Wave-Swept Environment. Princeton, NJ: Princeton University Press

[B7] Dirks J. H. Dürr V. (2011). Biomechanics of the stick insect antenna: damping properties and structural correlates of the cuticle. J. Mech. Behav. Biomed. Mater. 4, 2031–2042 10.1016/j.jmbbm.2011.07.002 22098903

[B8] Gewecke M. Heinzel H. G. (1980). Aerodynamic and mechanical properties of the antennae as air-current sense organs in *Locusta migratoria*. I. Static characteristics. J. Comp. Physiol. A 139, 357–366 10.1007/BF00610466

[B9] Göpfert M. C. Briegel H. Robert D. (1999). Mosquito hearing: sound-induced antennal vibrations in male and female *Aedes aegypti*. J. Exp. Biol. 202, 2727–2738 10504309 10.1242/jeb.202.20.2727

[B10] Haas F. Gorb S. Wootton R. J. (2000). Elastic joints in dermapteran hind wings: materials and wing folding. Arthropod Struct. Dev. 29, 137–146 10.1016/S1467-8039(00)00025-6 18088922

[B11] Haas F. Wootton R. J. (1996). Two basic mechanisms in insect wing folding. Proc. R. Soc. B Biol. Sci. 263, 1651–1658 10.1098/rspb.1996.0241

[B12] Heimbs S. Cichosz J. Klaus M. Kilchert S. Johnson A. F. (2009). Sandwich structures with textile-reinforced composite foldcores under impact loads. Compos. Struct. 92, 1485–1497 10.1016/j.compstruct.2009.11.001

[B13] Heinzel H. Gewecke M. (1987). Aerodynamic and mechanical properties of the antennae as air-current sense organs in *Locusta migratoria*. II. Dynamic characteristics. J. Comp. Physiol. A 161, 671–680 10.1007/BF00605008

[B14] Hepburn H. R. Chandler H. D. (1976). Material properties of arthropod cuticles: the arthrodial membranes. J. Comp. Physiol. 109, 177–198 10.1007/BF00689417

[B15] Honegger H. W. (1981). A preliminary note on a new optomotor response in crickets: antennal tracking of moving targets. J. Comp. Physiol. 142, 419–421 10.1007/BF00605454

[B16] Kier W. M. (2012). The diversity of hydrostatic skeletons. J. Exp. Biol. 215, 1247–1257 10.1242/jeb.056549 22442361

[B17] Kobayashi H. Daimaruya M. Vincent J. F. V. (1999). Effect of crease interval on unfolding manner of corrugated tree leaves. JSME Int. J. 42, 759–767 10.1299/jsmec.42.759

[B18] Kobayashi H. Kresling B. Vincent J. F. V. (1998). The geometry of unfolding tree leaves. Proc. R. Soc. B Biol. Sci. 265, 147–154 10.1098/rspb.1998.0276

[B19] Loudon C. (2005). Flexural stiffness of insect antennae. Am. Entomol. 51, 48–49

[B20] Loudon C. (2009). Antennae, in Encyclopedia of Insects, 2nd Edn., eds Resh V. E. Cardé R. (San Diego,CA: Elsevier, Academic Press Inc.), 21–23 10.1016/B978-0-12-374144-8.00006-0

[B21] Nijhout H. F. Sheffield H. G. (1979). Antennal hair erection in male mosquitoes: a new mechanical effector in insects. Science 206, 595–596 10.1126/science.40308 40308

[B22] Pass G. (1980). The anatomy and ultrastructure of the antennal circulatory organs in the cockchafer beetle *Melolontha melolontha* L. (Coleoptera, Scarabaeidae). Zoomorphology 96, 77–89 10.1007/BF00310078

[B23] Pass G. (1991). Antennal circulatory organs in Onychophora, Myriapoda and Hexapoda: functional morphology and evolutionary implications. Zoomorphology 110, 145–164 10.1007/BF01632871

[B24] Pass G. (2000). Accessory pulsatile organs: evolutionary innovations in insects. Annu. Rev. Entomol. 45, 495–518 10.1146/annurev.ento.45.1.495 10761587

[B25] Robinson W. H. (1996). Antennal grooming and movement behaviour in the German cockroach, *Blattella germanica* (L.), in Proceedings of the Second International Conference on Urban Pests, ed Wildey K. B. (Exeter, UK: Exeter Press), 361–369

[B26] Sandeman D. C. (1989). Physical properties, sensory receptors and tactile reflexes of the antenna of the australian freshwater crayfish *Cherax destructor*. J. Exp. Biol. 141, 197–217

[B27] Schenk M. Guest S. D. (2013). Geometry of Miura-folded metamaterials. Proc. Natl. Acad. Sci. U.S.A. 110, 3276–3281 10.1073/pnas.1217998110 23401549 PMC3587190

[B28] Snodgrass R. E. (1935). Principles of Insect Morphology. New York, NY: McGraw-Hill

[B29] Staudacher E. M. Gebhardt M. Dürr V. (2005). Antennal movements and mechanoreceptors: neurobiology of active tactile sensors. Adv. Insect Phys. 32, 49–205 10.1016/S0065-2806(05)32002-9

[B30] Vincent J. F. V. Wegst U. G. K. (2004). Design and mechanical properties of insect cuticle. Arthropod Struct. Dev. 33, 187–199 10.1016/j.asd.2004.05.006 18089034

[B31] Vogel S. (2013). Comparative Biomechanics: Life's Physical World. Princeton, NJ: Princeton University Press

[B32] Wainwright S. A. Biggs W. D. Currey J. D. Gosline J. M. (1976). Mechanical Design in Organisms. New York, NY: John Wiley & Sons

[B33] Wei Z. Y. Guo Z. V. Dudte L. Liang H. Y. Mahadevan L. (2013). Geometric properties of periodic pleated origami. Phys. Rev. Lett. 110, 1–5 10.1103/PhysRevLett.110.215501 23745895

[B34] Winder B. G. Magleby S. P. Howell L. L. (2009). Kinematic representations of pop-up paper mechanisms. J. Mech. Robot. 1, 1–10 10.1115/1.3046128

[B35] Zacharuk R. Y. (1985). Antennae and sensilla, in Comprehensive Insect Physiology Biochemistry and Pharmacology, 1st Edn., eds Kerkut G. A. Gilbert. L. I. (New York, NY: Pergamon Press), 1–69

